# The *Drosophila DmGluRA* is required for social interaction and memory

**DOI:** 10.3389/fphar.2013.00064

**Published:** 2013-05-23

**Authors:** Brian P. Schoenfeld, Richard J. Choi, Catherine H. Choi, Allison M. Terlizzi, Paul Hinchey, Maria Kollaros, Neal J. Ferrick, Eric Koenigsberg, David Ferreiro, David A. Leibelt, Steven J. Siegel, Aaron J. Bell, Thomas V. McDonald, Thomas A. Jongens, Sean M. J. McBride

**Affiliations:** ^1^Section of Molecular Cardiology, Department of Molecular Pharmacology and Medicine, Albert Einstein College of MedicineBronx, NY, USA; ^2^Department of Genetics, University of Pennsylvania School of MedicinePhiladelphia, PA, USA; ^3^Department of Medicine, Lehigh Valley Health SystemPA, USA; ^4^Department of Dermatology, Drexel University College of Medicine, PhiladelphiaPA, USA; ^5^Department of Psychiatry, University of Pennsylvania School of MedicinePhiladelphia, PA, USA

**Keywords:** mGluR, memory, *Drosophila*, long-term memory, *DmGluRA*, learning

## Abstract

Metabotropic glutamate receptors (mGluRs) have well-established roles in cognition and social behavior in mammals. Whether or not these roles have been conserved throughout evolution from invertebrate species is less clear. Mammals have eight mGluRs whereas *Drosophila* has a single *DmGluRA*, which has both Gi and Gq coupled signaling activity. We have utilized *Drosophila* to examine the role of *DmGluRA* in social behavior and various phases of memory. We have found that flies that are homozygous or heterozygous for loss of function mutations of *DmGluRA* have impaired social behavior in male *Drosophila*. Futhermore, flies that are heterozygous for loss of function mutations of *DmGluRA* have impaired learning during training, immediate-recall memory, short-term memory, and long-term memory as young adults. This work demonstrates a role for mGluR activity in both social behavior and memory in *Drosophila*.

## INTRODUCTION

The Metabotropic glutamate receptors (mGluRs) in mammals have been shown to be involved in memory formation, long-term depression (LTD), long-term potentiation in mammals and linked to autism spectrum disorders (ASDs) in humans (Serajee et al., 2003; Mukherjee and Manahan-Vaughan, 2012). In mammals there are eight mGluRs, which are divided into three groups. Canonically, previous studies have established that group I mGluRs in mammals activate the Gq pathway, while group II and group III mGluRs activate the Gi/Go signaling pathway (Niswender and Conn, 2010; Chaki et al., 2012; Mukherjee and Manahan-Vaughan, 2012). However, there is accumulating evidence that in mammals, due to promiscuity of coupling to Gi and Gq, group II mGluRs may activate the Gq signaling pathway and induce LTD in a manner dependent on phospholipase C (PLC) and inositol trisphosphate receptor (InsP3R) activity (Huang et al., 1997, 1999a,b; Otani et al., 1999, 2002), and group I mGluRs are capable of activating Gi (Kreibich et al., 2004). In *Drosophila* there is only one mGluR, *DmGluRA*, which is coupled to Gi and Gq signaling (McBride et al., 2005; Pan and Broadie, 2007; Pan et al., 2008; Gatto and Broadie, 2009; Tessier and Broadie, 2012). Gi coupled signaling is able to engage and activate both the PI3K and ERK signaling cascades as well as increase glycogen synthase kinase-3 beta (GSK-3beta) activity and Gq mediated mGluR activation is also able to activate GSK-3beta activity (Fan et al., 2004; Huang et al., 2006; Beaulieu et al., 2009; Yuskaitis et al., 2010).

*Drosophila DmGluRA* expression has been demonstrated in the brain including expression in areas critically involved in short-term memory such as the antennal lobes (ALs) and mushroom bodies (MBs; McBride et al., 1999; Zars et al., 2000; Yu et al., 2004; Pesavento et al., 2008) and long-term memory in the MBs (McBride et al., 1999; Pascual and Preat, 2001) where expression is particularly heavy (Ramaekers et al., 2001; Pan and Broadie, 2007; Pan et al., 2008). More recently a detailed analysis of DmGluRA protein expression in the central complex has been published, a region of the brain where the expression of several other metabotropic receptors implicated in mammalian learning was found (Kahsai et al., 2012). The MBs in the insect are thought to be analogous to the mammalian hippocampus as first postulated from structural similarity to the human hippocampus in 1850 by the French physiologist and anatomist DuJarin (Dujardin, 1850; Davis, 1993, 2011; Busto et al., 2010; Skoulakis and Grammenoudi, 2006). Additionally, DmGluRA protein has been demonstrated to play a role in signaling at the presynapse of the NMJ in *Drosophila* and therefore could be similarly affecting signaling at the presynapse in the brain (Pan and Broadie, 2007; Pan et al., 2008; Banerjee et al., 2010).

The role of *DmGluRA* in cognition has been previously demonstrated in studies of *Drosophila *models of Fragile X syndrome and Alzheimer’s disease. Fragile X is the leading inherited cause of intellectual disability and the leading known genetic cause of ASD. A fly model is based on loss of the of the fly *dfmr1* gene, the ortholog of the human *FMR1* gene, whose lack of expression leads to Fragile X syndrome. The Fragile X fly model has several behaviors in common with human symptoms including impairments in social interactions (Dockendorff et al., 2002) and cognitive impairments (McBride et al., 2005). Pharmacological blockade of the DmGluRA protein activity was able to rescue social interaction, immediate-recall memory and short-term memory in the Fragile X model representing the first time pharmacologic treatment rescued social impairments in an animal model of autism or memory impairments in an animal model of intellectual disability (Rubin, 1999b; McBride et al., 2005, 2012). Additionally, in this study, treatments initiated in development as well as those initiated in adulthood demonstrated efficacy in rescuing social interactions and memory.

*DmGluRA* has also been implicated in having a role in a *Drosophila* model of Alzheimer’s disease that is based on mutations of the *presenilin* gene (McBride et al., 2010). The underlying nature of signaling alterations arising from the mutations in *presenilin 1* or *presenilin 2* genes that give rise to familial Alzheimer’s disease (FAD) in humans are unclear (Saura et al., 2004; Walker et al., 2005; Qi-Takahara et al., 2005; Kumar-Singh et al., 2006; Sambamurti et al., 2006; De Strooper, 2007; Hardy, 2007; Isoo et al., 2007; Shen and Kelleher, 2007; Wolfe et al., 2007). Studies in model organisms indicate that the FAD-linked mutations lead to an impairment of *presenilin 1* or *presenilin 2* function (De Strooper, 2007; Kelleher and Purcell, 2008). This possibility suggests that some phenotypes associated with Alzheimer’s disease, including age-onset cognitive loss, may be attributable to a reduction in overall presenilin protein activity levels. In the *Drosophila* Alzheimer’s model young adult *Drosophila* (under 10 days of age, post-eclosion) have intact learning-during-training (LDT), immediate-recall memory and short-term memory, but have age dependent impairments in LDT and short-term memory at 30 days of age (McBride et al., 2010). Pharmacologic treatment with mGluR antagonists starting before cognitive impairments begin prevents cognitive impairment. Furthermore, treatment with mGluR antagonists starting after the onset of cognitive impairments reverses cognitive impairments in this model, indicating mGluR involvement in modulating synaptic plasticity well into adulthood (McBride et al., 2010). This indicates that in the Alzheimer’s fly model, just as in the Fragile X fly model over active mGluR activity is contributing to memory impairment. More recently, under active mGluR activity has been implicated in phenotypes exhibited by tuberous sclerosis type 2 model mice (Auerbach et al., 2011). In spite of these findings, the involvement of *DmGluRA* in social interactions and memory in otherwise normal flies has remained unexplored in *Drosophila*. The purpose of this study was to examine the role of *DmGluRA *in social interactions and memory in *Drosophila*.

## RESULTS

Social interaction can be examined in *Drosophila* in an ethologically relevant context by observing male courtship behavior directed toward female targets. Courting *Drosophila* males perform a characteristic sequence of behaviors: orienting toward and following the female, tapping her with his forelegs, vibrating one wing, licking her genitalia, and attempting to copulate (Bastock, 1955, 1956; Sturtevant, 1915). The percentage of time that the male spends performing any of these behaviors toward a target female during a defined period of time is referred to as the courtship index (CI; Siegel and Hall, 1979).

**FIGURE 1:**
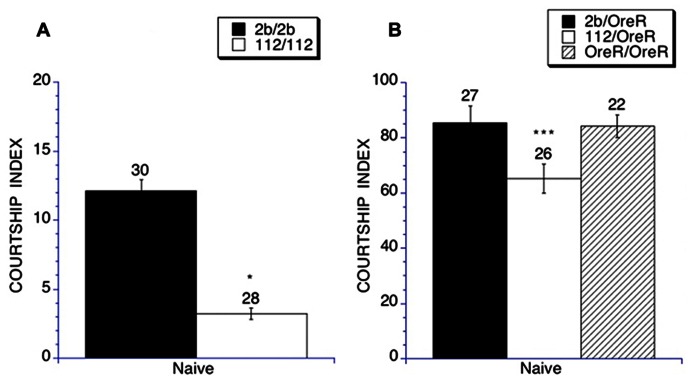
**Social interaction (Naïve courtship) is impaired in homozygous and heterozygous *DmGluRA*^*112*^ mutant flies**. Naïve courtship of *DmGluRA*, *DmGluRA*^*112*^ homozygous mutant flies, control *DmGluRA*^*2b*^ precise excision homozygous flies, *DmGluRA*^*112*^ heterozygous mutant flies, control *DmGluRA*^*2b*^ precise excision heterozygous flies and control OreR background flies were used. Panel **(A)** Filled black bars indicate control homozygous males (*DmGluRA*^*2b*^ precise excision, 2b/2b) and open bars indicate *DmGluRA*^*112*^ homozygous mutant males (112/112). Panel **(B)** Filled black bars indicate control heterozygous males (*DmGluRA*^*2b*^ precise excision, 2b/OreR); Open bars indicate *DmGluRA*^*112*^ heterozygous mutant males (112/OreR); Striped bars indicate OreR background males. Mean CIs (± SEM) are plotted; Ns are indicated above each bar for all groups. For levels of significance, *p < 0.01; ***p < 0.001. *DmGluRA*^*112*^ homozygous and heterozygous mutant males court virgin females less vigorously compared to control flies within the same genetic backgrounds.

We first examined the ability of young adult (6–10 days post-eclosion) homozygous null *DmGluRA*^*112*^ flies to perform naïve courtship with virgin female targets as well as the *DmGluRA*^*2b*^, a precise excision control line. We found courtship behavior to be significantly impaired in the *DmGluRA *homozygous mutant flies, with the flies demonstrating almost no courtship activity (CIs of 3.2 ± 0.4), whereas the genetic background control flies demonstrated intact courtship behavior (CIs of 12.1 ± 0.8; **Figure 1A**). This demonstrated that the *DmGluRA* activity is required for social interaction since there was a significant impairment in naïve courtship behavior compared to the control strain.

The low courtship activity of the *DmGluRA *mutants prevented us from examining memory in the homozygous mutant mGluR flies. Therefore to explore a possible role *of* *DmGluRA* in memory, we examined courtship and memory in flies heterozygous for this mutation. To do this we crossed both the *DmGluRA* null mutant (*DmGluRA*^*112*^) and precise excision control (*DmGluRA*^*2b*^) lines to Oregon R flies. We found that in the heterozygous state there was still an impairment in social interaction of the *DmGluRA*^*112*^ heterozygous flies compared to the *DmGluRA*^*2b*^ controls, 65.2 ± 5.3 vs 85.3 ± 6.2, again indicating a role for *DmGluRA* function in social interactions in *Drosophila* (**Figure 1B**). It should be noted that courtship in the OreR background is significantly higher than in the original background, mainly due to visual acuity differences in detecting motion, since the original background is white eyed. The *OreR/OreR* controls, the precise excision heterozygotes (*DmGluRA*^*2b*^/OreR) controls and the mutant heterozygotes (*DmGluRA*^*112*^/OreR) all had similar eye color. Furthermore, both control genotypes displayed similar levels of naïve courtship activity.

Although the heterozygous *DmGluRA* mutant flies displayed reduced naïve courtship activity, they still retained enough courtship activity to examine learning and various forms of memory using the conditioned courtship memory paradigm, an associative memory paradigm. In conditioned courtship, a male fly learns to modify his courtship behavior after experience with an unreceptive female (Siegel and Hall, 1979; Hall, 1994). Virgin females generally respond to a courting male by mating. However, recently mated females are unreceptive, display rejecting behaviors toward advances made by the male and have an overlapping but altered pheromonal profile that naïve males find less provocative than that of virgin female targets (Ejima et al., 2007). Normally, naïve male paired with a mated female target will initially court her, but his courtship activity soon decreases. This LDT is quantified, by comparing the CI during the first 10 min to the CI of the last 10 min period of a 1 h pairing with a previously mated female. In this paradigm wild-type flies typically show a ≥40% decrease in courtship activity (Joiner and Griffith, 1997; Kane et al., 1997). Hence, LDT is a form of behavioral plasticity but is distinct and separate from courtship suppression assayed after training, which is a form of associative memory (Tompkins et al., 1983; Ackerman and Siegel, 1986). When a male is paired with a virgin female after 1 h pairing experience with a mated female, his courtship remains depressed for 2–3 h (Siegel and Hall, 1979). This effect is not a general suppression of all courtship activity, because trained males do not modify their courtship of other pheromonally distinct targets (Ejima et al., 2005; Siwicki et al., 2005). After training with a mated female, memory is measured as a decrease in CI toward virgin females in trained males relative to naïve (sham trained) controls.

In *Drosophila*, five phases of memory have been elucidated by a combination of genetic and pharmacological dissection. There is an immediate-recall memory (immediate memory) at 0–2 min after training, short-term memory out to 1 h post-training, medium-term memory out to 6 h post-training, anesthesia-resistant memory out to 2 days post-training, and long-term memory lasting up to 9 days after training that appears to be dependent on protein synthesis (Skoulakis and Grammenoudi, 2006). Intact short-term memory is dependent on intact immediate recall. However, immediate recall and short-term memory are distinct from LDT. Therefore, intact memory can occur without LDT, and LDT can occur without post-training memory (Joiner and Griffith, 1997; Kane et al., 1997; McBride et al., 2005). Hence, in this study we chose to examine LDT, immediate-recall memory, short-term memory and long-term memory.

**FIGURE 2:**
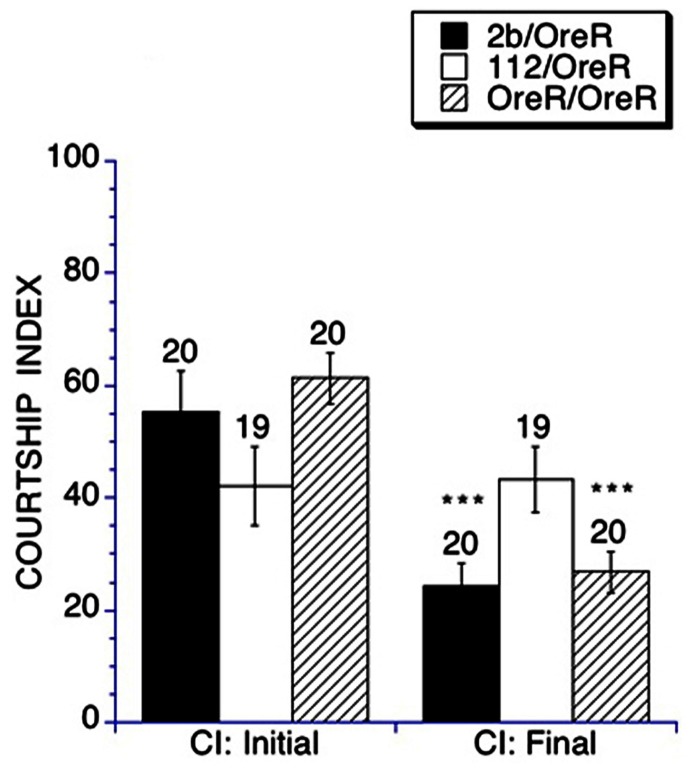
**The learning-during-training phase of conditioned courtship is impaired in heterozygous *DmGluRA*^*112*^ mutant flies**. Mean CIs (± SEM) are plotted; Ns are indicated above each bar for all groups. For levels of significance, ***p < 0.001. The initial and final courtship levels of control *DmGluRA*^*2b*^ precise excision heterozygous flies (filled black bars), *DmGluRA*^*112*^ heterozygous mutant flies (open bars) and OreR background flies (striped bars) are compared. Control *DmGluRA*^*2b*^ flies and control OreR background flies exhibited intact learning-during- training as demonstrated by a significant depression of courtship activity from the initial to the final interval of the training session, whereas heterozygous *DmGluRA*^*112*^ mutant flies did not demonstrate learning-during-training.

To assess LDT, a male fly was placed in a training chamber with a previously mated female for 1 h, and the amount of time the male spent courting in the initial 10 min interval was compared with the time spent engaged in courtship in the final 10 min interval. Heterozygous *DmGluRA*^*112*^ mutants display impaired LDT as young adults (**Figure 2**), similar to what he been previously observed in older *DmGluRA*^*112*^ mutant flies at 30 days of age (McBride et al., 2010). In contrast heterozygous *DmGluRA*^*2b*^ controls and the *OreR/OreR* controls displayed intact LDT. This demonstrates a requirement for *DmGluRA* function in LDT.

**FIGURE 3:**
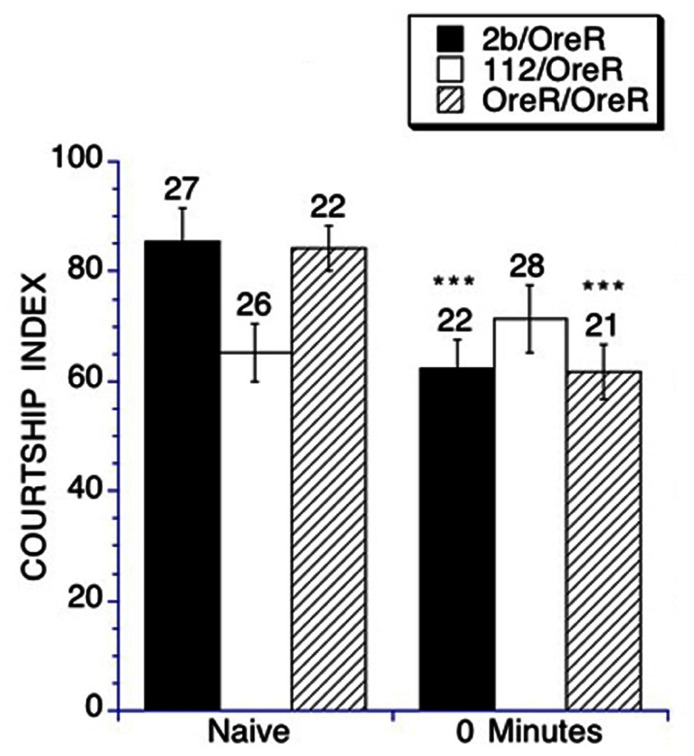
**Immediate-recall memory of conditioned courtship is impaired in heterozygous *DmGluRA*^*112*^ mutant flies**. Immediate-term memory was measured by placing a trained male in a holding chamber for 0–2 min after training, then subsequently placing him in a testing chamber with a virgin female target for a 10 min courtship interval. The resulting CI is compared to the CI obtained for naïve courtship. Mean CIs (± SEM) are plotted; Ns are indicated above each bar for all groups. For levels of significance, ***p < 0.001. Control *DmGluRA*^*2b*^ flies (filled black bars) and control OreR background flies (striped bars) exhibited intact immediate-recall memory as demonstrated by a significant depression of courtship activity in the trained versus the naïve groups. The *DmGluRA*^*112*^ mutant flies (open bars) did not demonstrate a suppression of courtship activity after training and therefore had impaired immediate-recall memory.

To assess immediate-recall memory, a male fly was placed in a training chamber with a previously mated female for 1 h, and subsequently paired with a virgin female within 2 min of completing training. A lower CI compared to naïve trained (untrained) flies is indicative of memory. Heterozygous *DmGluRA*^*112*^ mutants display impaired immediate-recall memory as young adults, as they are not able to suppress their courtship upon subsequent pairing with a virgin female target (**Figure 3**). In contrast heterozygous *DmGluRA*^*2b*^ controls and the *OreR/OreR* controls displayed intact immediate-recall memory. This demonstrates a requirement for *DmGluRA* function in immediate-recall memory.

**FIGURE 4:**
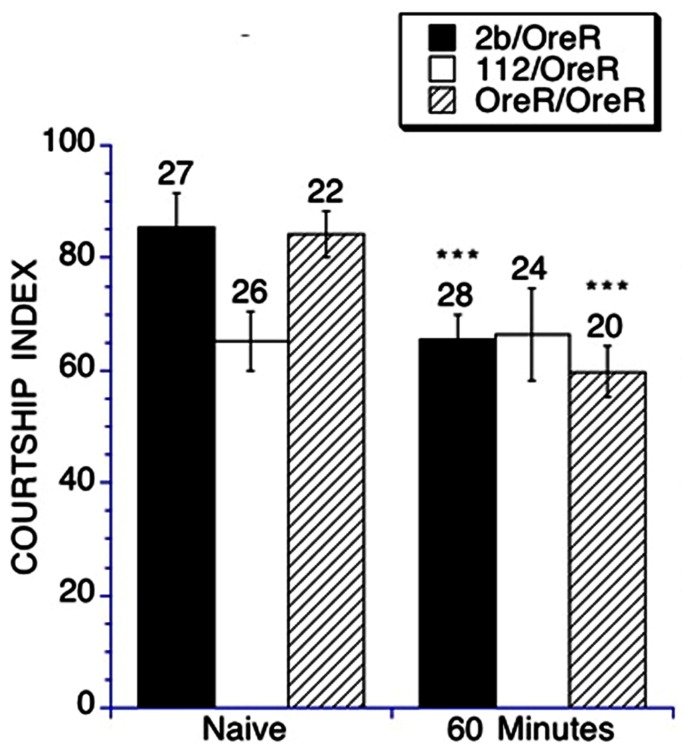
**The short-term memory of conditioned courtship is impaired in heterozygous *DmGluRA*^*112*^ mutant flies**. Short-term memory was measured by placing a trained male in a holding chamber for 60 min, then subsequently placing him in a testing chamber with a virgin female target for a 10 min courtship interval. The resulting CI is compared to the CI obtained for naïve courtship. Mean CIs ( ± SEM) are plotted; Ns are indicated above each bar for all groups. For levels of significance, ***p < 0.001. Control *DmGluRA*^*2b*^ flies (filled black bars) and control OreR background flies (striped bars) exhibited intact short-term memory as demonstrated by a significant depression of courtship activity in the trained versus the naïve groups. The *DmGluRA*^*112*^ mutant flies (open bars) did not demonstrate a suppression of courtship activity after training and therefore had impaired short-term memory.

To assess short-term memory, a male fly was placed in a training chamber with a previously mated female for 1 h, and subsequently paired with a virgin female 60 min after completing training. A lower CI compared to naïve-trained flies is indicative of memory. Heterozygous *DmGluRA*^*112*^ mutants do not demonstrate a suppression of their courtship upon subsequent pairing with a virgin female target, therefore they do not demonstrate short-term memory (**Figure 4**). In contrast heterozygous *DmGluRA*^*2b*^ controls and the *OreR/OreR* controls displayed a suppression of courtship after training and therefore demonstrated short-term memory. This demonstrates a requirement for *DmGluRA* function in short-term memory.

**FIGURE 5:**
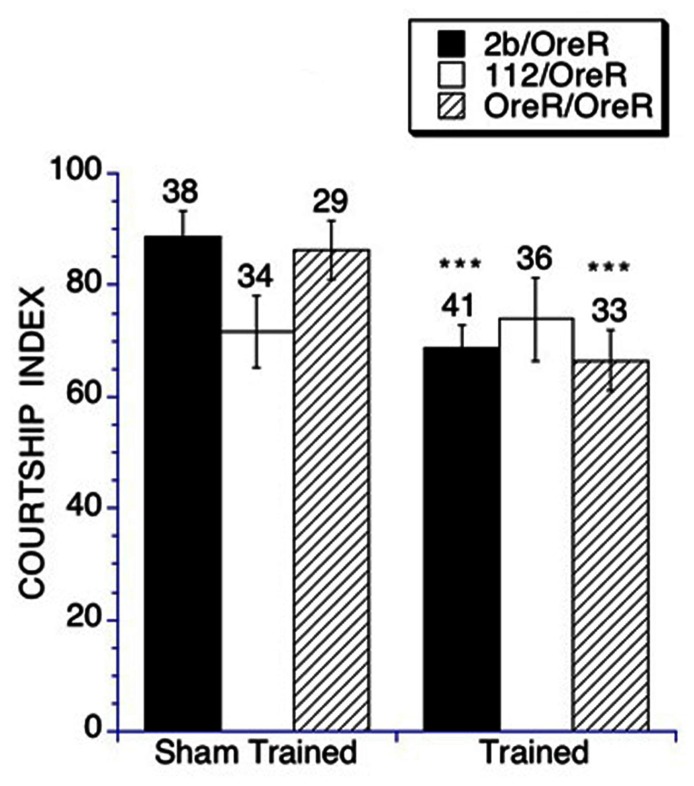
**The long-term memory of conditioned courtship is impaired in heterozygous *DmGluRA*^*112*^ mutant flies**. For long-term memory, males were either paired with a non-receptive female for 7 h or sham trained (also referred to as naïve trained) in the absence of a female for the same period. The males from both training classes were then kept in isolation for 4 days then paired with a virgin female, and monitored for courtship activity. The resulting CI after training is compared to the CI obtained for naïve courtship. Mean CIs (± SEM) are plotted; Ns are indicated above each bar for all groups. For levels of significance, ***p < 0.001. Control *DmGluRA*^*2b*^ flies (filled black bars) and control OreR background flies (striped bars) exhibited intact long-term memory as demonstrated by a significant depression of courtship activity in the trained versus the naïve groups. The *DmGluRA*^*112*^ mutant flies (open bars) did not demonstrate a suppression of courtship activity after training and therefore had impaired long-term memory.

Finally we examined if *DmGluRA* function was required for long-term memory (McBride et al., 1999; Banerjee et al., 2010). To assess long-term memory, a male fly was placed in a training chamber containing food with a previously mated female for 7 h, and subsequently paired with a virgin female 4 days after completing training (McBride et al., 1999; Banerjee et al., 2010). Again, a lower CI compared to sham trained (naïve-trained) flies is indicative of memory. Heterozygous *DmGluRA*^*112*^ mutants do not demonstrate a suppression of their courtship upon subsequent pairing with a virgin female target, therefore they do not demonstrate long-term memory (**Figure 5**). In contrast heterozygous *DmGluRA*^*2b*^ controls and the *OreR/OreR* controls displayed a suppression of courtship after training and therefore demonstrated long-term memory. This demonstrates a requirement for *DmGluRA* function in the formation of long-term memory.

**FIGURE 6:**
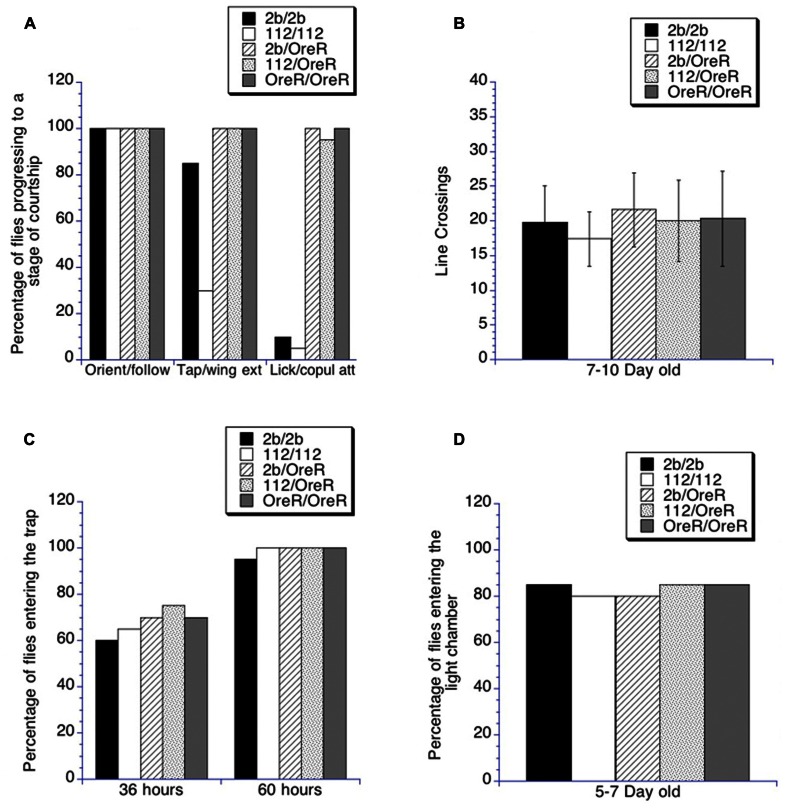
**Analysis of courtship quality, locomotor activity, olfaction, and visual acuity in *DmGluRA* mutants. (A–D)** The Ns for all genotypes in all panels is >19. Filled black bars indicate control homozygous males (*DmGluRA*^*2b*^ precise excision, 2b/2b); Open bars indicate *DmGluRA*^*112*^ homozygous mutant males (112/112); Striped bars indicate control heterozygous males (*DmGluRA*^*2b*^ precise excision, 2b/OreR); Stippled bars indicate *DmGluRA*^*112*^ heterozygous mutant males (112/OreR); Gray bars indicate OreR background males. **(A)** The quality of courtship that was performed by naïve males was further analyzed by binning the number of males that advanced to particular phases of courtship for each genotype that was shown in **Figure 1**. All of the genotypes demonstrated that they could perform each phase of courtship. The *DmGluRA*^*112*^ homozygous mutant males (112/112) did not demonstrated as much wing extension as the control males (*p* < 0.05) and neither the homozygous control males (*DmGluRA*^*2b*^ precise excision, 2b/2b) or the *DmGluRA*^*112*^ homozygous mutant males (112/112) progressed to the final stage of courtship as often as the control heterozygous males (*DmGluRA*^*2b*^ precise excision, 2b/OreR), *DmGluRA*^*112*^ heterozygous mutant males (112/OreR), or the OreR background males (*p* < 0.05). **(B)** Locomotor activity was measured by a line crossing assay (Griffith et al., 1993; McBride et al., 2005). Mean CIs (± SEM) are plotted; No significant differences were detected between any of the genotypes. **(C)** To measure olfactory capabilities we used the olfactory trap assay (Orgad et al., 2000; McBride et al., 2005). No differences were found between any of the genotypes tested with this assay at the 36 or 60 h time points. **(D)** To examine the visual capabilities of the genotypes and various treatment groups, we used the Y maze test (Orgad et al., 2000; McBride et al., 2005). No significant difference in the ability of the flies to detect light was observed in any of the genotypes.

To ensure that the decreased courtship activity of the homozygous and heterozygous *DmGluRA*^*112*^ mutants was not the result of specific impairment in not being able to complete the various phases of courtship, we measured the percentage of flies that progressed through the stages of courtship. The homozygous *DmGluRA*^*112*^ mutants demonstrated the ability to progress through all of the stages of courtship in a 10 min testing period. Both the homozygous *DmGluRA*^*112*^ mutants and the homozygous control precise excision *DmGluRA*^*2b*^ had a significantly lower percentage of flies progressing to the licking/attempted copulation stage compared to the flies that were crossed to the OreR background (*p* < 0.05 by chi square). However, the *DmGluRA*^*112*^ mutants and the homozygous control precise excision *DmGluRA*^*2b*^ did not differ from each other in the percentage of flies that reached this final step (**Figure 6A**). Both of the heterozygous lines as well as the OreR background control reached similar percentages of achieving all stages of courtship (**Figure 6A**). Since both the homozygous and heterozygous *DmGluRA*^*112*^ mutant flies were observed to be capable of performing all of the steps of courtship, the lack of courtship activity does not appear to be secondary to some type of impairment that is rendering them incapable of completing all of the steps of courtship behavior. To ensure that the decreased courtship activity of the homozygous and heterozygous *DmGluRA*^*112*^ mutants was not the result of locomotor activity impairments, we examined locomotor function in the dishes utilized for the conditioned courtship testing (McBride et al., 2005, 2010). We did not find differences in spontaneous line crossing between homozygous or heterozygous *DmGluRA*^*112*^ mutant flies vs homozygous or heterozygous *DmGluRA*^*2b*^ control flies or the *OreR/OreR* control flies (**Figure 6B**). Additionally neither the homozygous or heterozygous flies in any of the genotypes displayed gross impairments in olfaction or vision (**Figures 6C,D**).

## DISCUSSION

Although a role for mGluRs signaling is well established in memory processes in mammals, the role of the only *Drosophila* mGluR, *DmGluRA*, has remained relatively unstudied with regard to social interaction or memory (Serajee et al., 2003; Mukherjee and Manahan-Vaughan, 2012). The expression of *DmGluRA* in the *Drosophila* brain is in areas critically involved in social behavior and memory including the ALs and the MBs, thus may contribute to these behavioral and cognitive processes (Ramaekers et al., 2001; Pan and Broadie, 2007; Pan et al., 2008).

Herein, we demonstrate that the *DmGluRA* activity is required for social interaction. We found that in the homozygous and heterozygous *DmGluRA*^*112*^ mutants have impairments in social behavior. This data fits well with the previous finding that a mutation in mGluR8 is linked to autism in humans (Serajee et al., 2003). Heterozygous *DmGluRA*^*112*^ mutants display impaired learning during training as young adults, thereby demonstrating a requirement for *DmGluRA* function in LDT, which may be analogous to working memory in mammals. Also heterozygous *DmGluRA*^*112*^ mutants display impairments in immediate-recall-memory, short-term memory, and long-term memory as young adults. These findings fit well with the known role of mGluRs in short-term and long-term memory formation in mammals (Mukherjee and Manahan-Vaughan, 2012). In our study the deficits in social behavior and memory did not appear to be caused by impairments in vision, olfaction, locomotion, or the capability to perform complicated coordinated motor tasks such as copulation or flight, thus *DmGluRA *function appears to be specific for social and cognitive tasks.

This study illustrates an evolutionarily conserved role of the mGluRs in synaptic plasticity and memory formation which is an important finding in the context of using lower organisms to model cognitive diseases such as Fragile X or Alzheimer’s disease. Indeed, these are two disease models where antagonizing the *Drosophila DmGluRA* has been demonstrated to rescue social and/or memory impairments (McBride et al., 2005, 2010; Choi et al., 2010). It was in the *Drosophila* model of Fragile X that pharmacological blockage of DmGluRA protein function was first demonstrated to rescue social interaction, immediate-recall memory and short-term memory representing the first time pharmacologic treatment rescued social impairments in an animal model of autism or memory impairments in an animal model of intellectual disability (Rubin, 1999b; McBride et al., 2005, 2012). Additionally, it was demonstrated that treatments initiated in development as well as in adulthood demonstrated efficacy in rescuing social interactions and memory. The finding that adulthood treatments could ameliorate phenotypes associated with developmental disorders was paradigm shifting (Rubin, 1999a; State, 2010) and has now been demonstrated in other models of developmental disorders (Rubin, 1999c; Li et al., 2005; Guy et al., 2007). This strategy of decreasing mGluR activity to rescue cognition in the Fragile X model was later confirmed in mouse model of Fragile X by genetic and pharmacologic manipulation (Yan et al., 2005; Dolen et al., 2007; Choi et al., 2011) and has met with some early success in trials with Fragile X patients (Jacquemont et al., 2011). This demonstrates the important role of *DmGluRA* function not just in development, but also in adulthood, warranting additional studies.

In *Drosophila* or cell culture models of Alzheimer’s disease antagonizing mGluRs has been efficacious in rescuing phenotypes associated with the models including cognitive impairment and Abeta secretion (Kim et al., 2010; McBride et al., 2010). Furthermore, both agonist and antagonists of mGluRs are under development for the treatment of depression in humans (Chaki et al., 2012; Mukherjee and Manahan-Vaughan, 2012). The current work along with the extensive mammalian literature on the involvement of mGluRs in memory illustrate that caution should be observed when looking at the mGluRs as receptors to modulate for the rescue of disease specific symptoms, because they may have unwanted effects on other aspects of cognition.

At first pass our results demonstrating that reduction of *DmGluRA* activity negatively impacts social behavior and cognition may seem counterintuitive, because antagonism of this receptors signaling can enhance memory in specific disease models. First, we have previously found that treatment with mGluR antagonists does not enhance memory in wild-type flies, indeed they impair memory and social activity (McBride et al., 2005; Choi et al., 2010). Also, toward this point it is important to keep in mind the way the molecular signaling occurs during memory formation. *DmGluRA* is predominantly coupled to Gi, thereby suppressing cAMP signaling. There is well documented literature in the fly field that indicates that cognition is impaired if cAMP levels are either too high, or too low. The *dnc* mutant has severe memory impairments and results from too much cAMP. The *dnc* mutation would be analogous to the *DmGluRA* mutants, with too much cAMP. In contrast the *rut* mutation leads to too little cAMP and also results in memory impairment (Skoulakis and Grammenoudi, 2006). This would be analogous to the fly models of Alzheimer’s disease and fragile X syndrome, where the problem is too little cAMP and it is corrected by treatment with mGluR antagonists which should correct the cAMP to a level were normal memory can occur (McBride et al., 2005, 2010; Choi et al., 2010, 2011).

In conclusion, this work demonstrates that in *Drosophila*, just as in mammals, proper *DmGluRA* function is required for social behavior and various aspects of cognition including LDT, immediate-recall memory, short-term memory, and long-term memory.

## MATERIALS AND METHODS

### BEHAVIORAL TRAINING AND TESTING

Virgin male flies were collected under ether anesthesia within 4 h of eclosion. Virgin XX, y, f (attached X) females were collected on the day of eclosion and kept in food vials in groups of 10–15. Flies were aged in a 12 h light/dark cycle before behavioral training and testing. All testing was performed during the relative light phase. Mated females were 5 days old and observed to mate with a male the night before training. The virgin females that were used as targets were 4 days old (Joiner and Griffith, 1997; McBride et al., 1999, 2005).

For courtship behavior testing, males of the appropriate genotypes were collected within 4 h of eclosion and kept in isolation before testing. All flies were kept in 12 h light/dark cycles at 25°C and 50–75% relative humidity and were aged 6–10 days post-eclosion before training. All male subjects were transferred to fresh control food the day before testing (McBride et al., 1999, 2005, 2010). Male flies were assigned to random groups for behavior training and testing, which was performed blind (Siegel and Hall, 1979; Kane et al., 1997; McBride et al., 1999). The total amount of time a male was engaged in courtship activity while paired with an unanesthetized target female during a test period of 10 min or until successful copulation occurred was scored. A CI was calculated as the percentage of total observation time spent courting (Siegel and Hall, 1979). Testing of naïve courtship, LDT, immediate-recall and short-term memory were performed as previously described (McBride et al., 1999, 2005). For the naïve courtship analysis, the male was sham trained for 1 h in the training chamber without the addition of the female. The male was then transferred to the mating chamber containing a virgin female. Males were monitored for courtship activity that included orienting, following of the female, wing extension and vibration, tapping of female with his foreleg, genital licking and attempted copulation for a period of 10 min, or until copulation occurred.

Measurement of immediate-recall was made by pairing a naïve male with a non-receptive female for a single 1 h training session and then placing him in a second chamber with a receptive female within 2 min of completing training. Short-term memory was assessed by taking a male that had been trained with a non-receptive female for 1 h and placing him in isolation for 1 h before pairing with a virgin, receptive, female. At least 16 animals were tested for each genotype during analyses of naïve courtship, learning during training, immediate recall, short-term memory, and long-term memory.

The training paradigm for assessment of long-term memory is derived from McBride et al. (McBride et al., 1999; Banerjee et al., 2010). Males were paired with a non-receptive female for seven continuous hours and then kept in isolation for 4 days before testing. Sham-trained males were treated identically, except for the exposure to the training female. The observers were blind to the genotypes of the animals for all courtship studies (Banerjee et al., 2010; Sidyelyeva et al., 2010). Locomotor, olfaction, and visual acuity testing was done as in the study by McBride et al. (Griffith et al., 1993; McBride et al., 2005; Orgad et al., 2000).

### *Drosophila* Strains

The *Drosophila* strains were cultured as in the study by McBride et al. (2005). The DmGluRA mutant and control lines used during this study are white eyed and have been previously described and utilized, they are the previously described null allele of *DmGluRA* (*DmGluRA*^*112*^) and precise excision wild-type allele (*DmGluRA*^*2b*^) that provides an appropriate background control for the null allele (Bogdanik et al., 2004). Heterozygous versions of the mutant and control were obtained by crossing males to Oregon R virgin females. Heterozygous *DmGluRA*^*112*^ and *DmGluRA*^*2b*^ F1 males were selected from the resultant progeny (McBride et al., 2010). The *DmGluRA* locus is on the 4th chromosome.

### STATISTICAL ANALYSES

Courtship index of tested males were subjected to arcsin square root transformations to approximate normal distributions since not all of the sets of data were normal distributions, as is common in conditioned courtship data sets (Joiner and Griffith, 1997; McBride et al., 1999, 2005). For statistical comparison between the genotypes and treatments, Two Way ANOVA was used for genotype and treatment, with genotype resulting in a *p* value of 0.0001 and treatment resulting in a *p* value of 0.0001. The interaction *p* value was 0.0001. The *post hoc* analysis used for the comparison for the figures was the Bonferroni analysis (Villella and Hall, 1996; McBride et al., 1999, 2005; Ejima et al., 2007). For the figures we have placed asterisks according to the *post hoc* Bonferroni analysis since this is demonstrating memory or no memory within a specific genotype according to the provided experience (treatment) of the flies (Villella and Hall, 1996; McBride et al., 1999, 2005, 2010; Ejima et al., 2007). For line crossing experiments standard student *t* test was used and for **Figure 6** binning analysis, olfaction and vision studies chi squared analysis was performed (McBride et al., 2005). All statistics were performed using both Statview 3.0 and Prism 5.0.

## Conflict of Interest Statement

The authors declare that the research was conducted in the absence of any commercial or financial relationships that could be construed as a potential conflict of interest.
